# Protective Effect of Baicalin against *Clostridioides difficile* Infection in Mice

**DOI:** 10.3390/antibiotics10080926

**Published:** 2021-07-30

**Authors:** Abraham Joseph Pellissery, Poonam Gopika Vinayamohan, Deepa Ashwarya Kuttappan, Neha Mishra, Breno de Oliveira Fragomeni, Kendra Maas, Shankumar Mooyottu, Kumar Venkitanarayanan

**Affiliations:** 1 Department of Animal Science, University of Connecticut, Storrs, CT 06269, USA; abraham.pellissery@uconn.edu (A.J.P.); deepa.kuttappan@uconn.edu (D.A.K.); breno.fragomeni@uconn.edu (B.O.F.); 2 Department of Veterinary Preventive Medicine, Ohio State University, Columbus, OH 43210, USA; vinayamohan.1@osu.edu; 3 Department of Pathobiology and Veterinary Science, University of Connecticut, Storrs, CT 06269, USA; neha.mishra@uconn.edu; 4 Microbial Analysis, Resources, and Services, University of Connecticut, Storrs, CT 06269, USA; kendra.maas@uconn.edu; 5 Department of Veterinary Pathology, Iowa State University, Ames, IA 50011, USA; shaan@iastate.edu

**Keywords:** *Clostridioides difficile*, baicalin, microbiome, gut dysbiosis, mouse model

## Abstract

This study investigated the prophylactic and therapeutic efficacies of baicalin (BC), a plant-derived flavone glycoside, in reducing the severity of *Clostridioides difficile* infection (CDI) in a mouse model. In the prophylactic trial, C57BL/6 mice were provided with BC (0, 11, and 22 mg/L in drinking water) from 12 days before *C. difficile* challenge through the end of the experiment, whereas BC administration started day 1 post challenge in the therapeutic trial. Both challenge and control groups were infected with 10^6^ CFU/mL of hypervirulent *C. difficile* BAA 1803 spores or sterile PBS, and the clinical and diarrheal scores were recorded for 10 days post challenge. On day 2 post challenge, fecal and tissue samples were collected from mice prophylactically administered with BC for microbiome and histopathologic analysis. Both prophylactic and therapeutic supplementation of BC significantly reduced the severity of colonic lesions and improved CDI clinical progression and outcome compared with control (*p* < 0.05). Microbiome analysis revealed a significant increase in Gammaproteobacteria and reduction in the abundance of protective microbiota (Firmicutes) in antibiotic-treated and *C. difficile*-infected mice compared with controls (*p* < 0.05). However, baicalin supplementation favorably altered the microbiome composition, as revealed by an increased abundance in beneficial bacteria, especially *Lachnospiraceae* and *Akkermansia*. Our results warrant follow-up investigations on the use of BC as an adjunct to antibiotic therapy to control gut dysbiosis and reduce *C. difficile* infection in humans.

## 1. Introduction

*Clostridioidesdifficile* is an important cause of nosocomial, antibiotic-associated diarrhea around the world [1,2]. The pathogen causes a toxin-mediated colitis in individuals of all age groups, with more severity observed in elderly and immunocompromised patients [3]. In the United States, more than 453,000 cases of *C. difficile* infection (CDI) with 29,000 deaths are reported annually, which incur an economic burden ranging between USD 0.4 to 3.0 billion as healthcare-associated costs [4,5]. The increased incidence of CDI in humans is primarily attributed to the emergence of NAP1/ribotype 027, a highly toxigenic and hypervirulent *C. difficile* strain [1,6,7,8].

Generally, individuals requiring long-term antibiotic therapy and gastric acid suppressing agents are highly predisposed to CDI [9,10,11]. Broad-spectrum antibiotics and acid suppressants alter the diversity and abundance of the normal gut microbial communities, resulting in a condition known as gut dysbiosis [12,13,14,15]. The dysbiotic gut environment facilitates *C. difficile* spore germination, outgrowth, colonization, and toxin productions in the distal gut [16]. *C. difficile* exotoxins—namely, toxin A and toxin B—disrupt the actin cytoskeleton and interepithelial tight junctions of the colonic epithelium, leading to severe diarrhea and suppurative inflammation that could culminate in pseudomembranous colitis and toxic megacolon in extreme cases [1,8,17,18].

Although extended antibiotic therapy predisposes individuals to CDI, antibiotics are still considered as the primary line of treatment for this disease, and the most commonly prescribed drugs include metronidazole, vancomycin, and fidaxomicin [4,7,19,20]. However, *C. difficile* has been continuously acquiring resistance to different classes of antibiotics, including those currently in clinical use against CDI [7,21]. With global emergence of antibiotic resistant and hypervirulent *C. difficile* strains, the Centers for Disease Control and Prevention (CDC) categorized the pathogen a few years ago as one among the three urgent threats to public health [22]. Therefore, there is an emergent need to identify alternative therapeutic agents that could reduce *C. difficile* virulence without adversely affecting the gut microbiota.

Phytochemicals represent a natural group of molecules that have been used for treating various diseases in traditional medicine [23]. Baicalin (5,6-dihydroxy-7-O-glucuronide flavone) is a flavone glycoside present in the plant *Scutellaria baicalensis* Georgi, known to possess antimicrobial, antioxidant, and anti-inflammatory properties [24,25,26,27,28,29,30]. Previously, our laboratory demonstrated the use of baicalin as a potential anti-*C. difficile* therapeutic agent owing to its inhibitory effect on *C. difficile* toxin production with minimal effects on the growth of selected beneficial microbiota in vitro [31]. As a logical next step, this study investigated the prophylactic and therapeutic effects of baicalin against CDI in an in vivo model by focusing on the clinical course and host microbiome changes in mice. Mouse models for CDI are well established, and antibiotic-induced gut dysbiosis in mice can be simulated by administering antibiotics orally and intraperitoneally, followed by inoculation of *C. difficile* spores [32,33].

## 2. Results

### 2.1. Effect of Baicalin Supplementation on the Incidence of Diarrhea and Severity of C. difficile Infection in Mice

**Figure 1:**
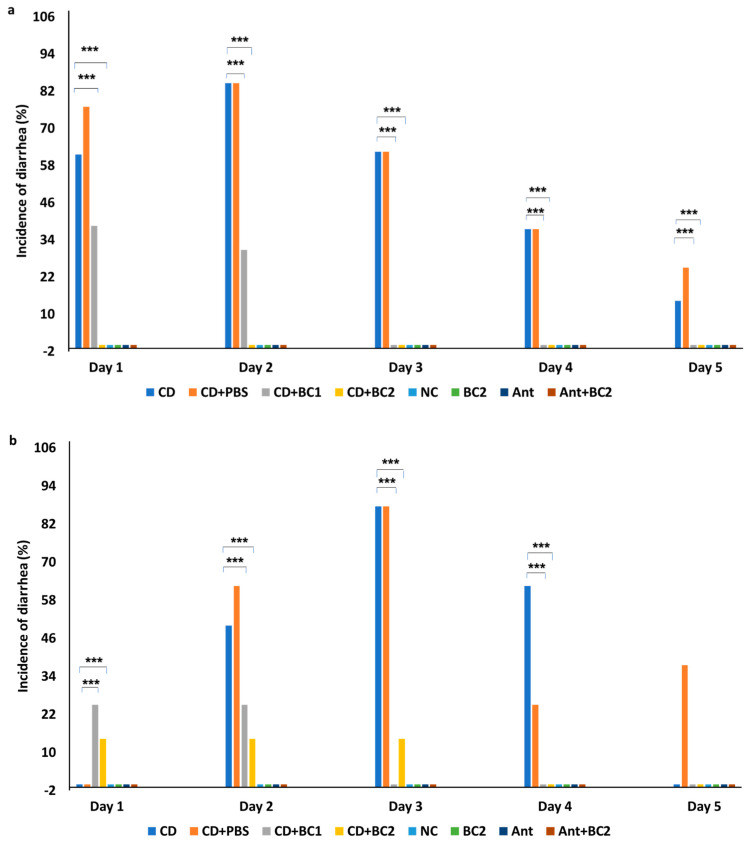
Effect of baicalin supplementation on the incidence of diarrhea in mice after CDI. Percentage incidence of diarrhea was recorded from 1 DPI to 5 DPI in the different treatment groups in the prophylactic BC study (**a**) therapeutic BC study (**b**). *** indicates a statistically significant difference (*p* < 0.0001) relative to challenged, positive control (CD) vs. the baicalin-treated challenged mice (CD+BC1 or CD+BC2). Error bars represent SEM. Treatment groups: NC (unchallenged negative control), Ant (unchallenged antibiotic control), Ant+BC (unchallenged antibiotic + 22 mg/L BC control), BC2 (unchallenged 22 mg/L BC control), CD (Ant+*C. difficile*-challenged control), CD+PBS (Ant+*C. difficile*-challenged control, PBS solvent control), CD+BC1 (Ant+ CD + 11 mg/L BC), CD+BC2 (Ant+ CD + 22 mg/L BC).

The prophylactic efficacy of baicalin against CDI in mice was assessed by supplementing the phytochemical in drinking water at two different concentrations (11 and 22 mg/L). Oral administration of 10^6^ CFU/mL *C. difficile* spores (ATCC BAA 1803) resulted in high morbidity with low mortality in infected mice. In *C. difficile*-infected control groups (CD), 61% and 85% of animals showed severe diarrhea on the first- and second-day post infection (DPI) (*n* = 13), respectively (Figure 1a). On 7 DPI, one animal from the CD group died, and no further mortality was recorded in this group (Supplementary Figure S3a). Although diarrhea continued for five days in the CD group, there was no increase in the percentage incidence of diarrhea thereafter (data not shown). However, the incidence of diarrhea was significantly lower in the CD+BC1 (challenged mice treated with 11 mg/L of BC) group, with 38% and 31% incidence on 1 DPI and 2 DPI, respectively, and with the absence of diarrhea on the subsequent days (Figure 1a). Moreover, diarrhea was not observed in the CD+BC2 group (challenged mice treated with 22 mg/L of BC) (*p* < 0.05), although there were two mortalities recorded in this group, one each on days 2 and 3 post infection (Supplementary Figure S3a).

In the therapeutic trial, baicalin was supplemented in drinking water similar to the prophylactic trial but was initiated from day 1 post challenge. Interestingly, the *C. difficile* positive control group (Ant+CD) did not show any diarrhea on 1 DPI; however, diarrhea was observed in 62.5% and 87.5% animals on 2 DPI (*n* = 13) and 3 DPI (*n* = 5), respectively (Figure 1b). Diarrhea was observed until the fifth day post infection in this group, with no additional increase in percentage incidence after 3 DPI. Diarrhea was observed from 1 DPI in the CD+BC1 and CD+BC2 groups, although a significantly reduced incidence was observed for both BC-treated groups compared with the positive control (*p* < 0.05). An incidence of 25% was observed on both 2 DPI and 3 DPI in CD+BC1 group, with no diarrhea thereafter. In the CD+BC2 group, the incidence of diarrhea stayed at 14% for days 1–3 post infection, with no more diarrhea observed for the remainder of experiment duration (Figure 1b). In addition, there was only one mortality recorded in the *C. difficile*-positive control group on 6 DPI (Supplementary Figure S3b).

No diarrhea was observed in the control groups (i.e., negative control (NC), baicalin control (BC2), antibiotic control (Ant), and antibiotic with baicalin control (Ant+BC2)) in both the prophylactic and therapeutic BC studies.

### 2.2. Effect of Baicalin Supplementation on Clinical Score and Body Weight of C. difficile-Infected Mice

**Figure 2:**
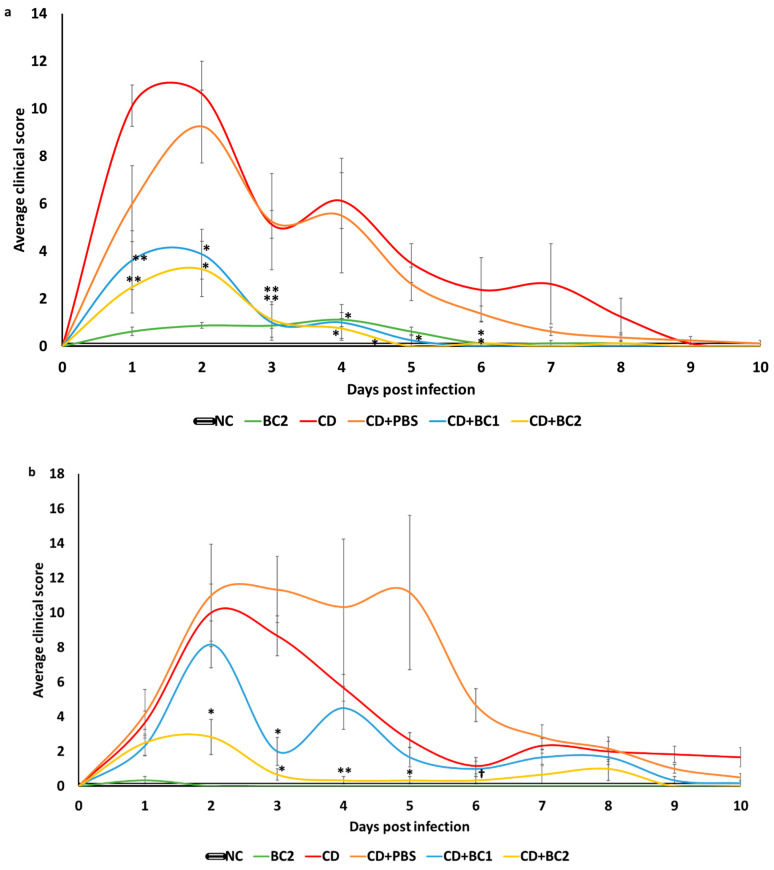

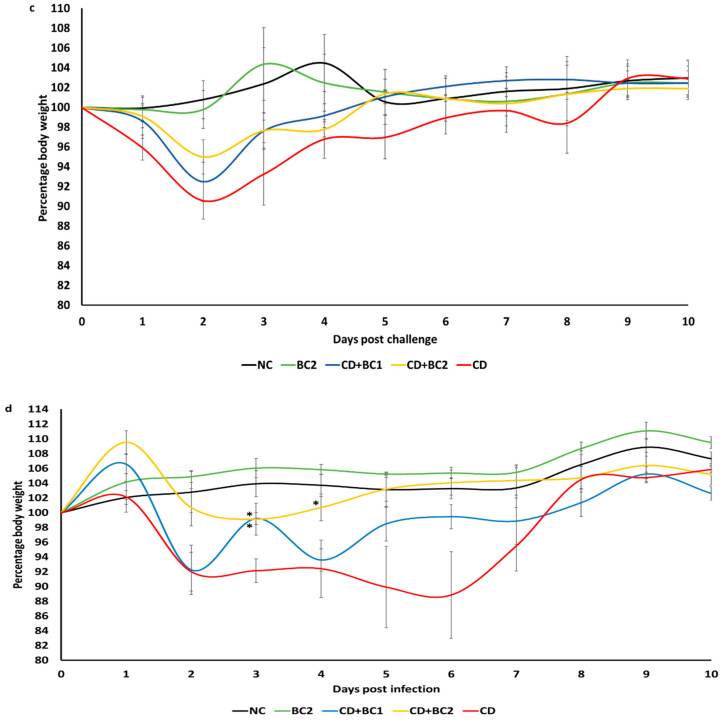
Effect of baicalin supplementation on clinical severity and change in body weight in mice after CDI. Average clinical scores and percentage body weights were recorded from 1 DPI to 10 DPI for the different treatment groups in the prophylactic BC study (**a**,**c**) and therapeutic BC study (**b**,**d**). **, * indicates a statistically significant difference (*p* < 0.001, *p* < 0.05, respectively) relative to the untreated challenge group (CD); † symbol indicates a statistically significant difference (*p* < 0.05) relative to CD+PBS. Percentage body weights among treatment groups (in (c,d) were compared within the same day time point. Error bars represent SEM. Treatment groups: NC (unchallenged negative control), Ant (unchallenged antibiotic control), Ant+BC (unchallenged antibiotic + 22 mg/L BC control), BC2 (unchallenged 22 mg/L BC control), CD (Ant+*C. difficile*-challenged control), CD+PBS (Ant+*C. difficile*-challenged control, PBS solvent control), CD+BC1 (Ant+CD + 11 mg/L BC), CD+BC2 (Ant+CD + 22 mg/L BC).

Clinical scores of animals from different treatment groups were individually recorded using a standard score chart, from 1 DPI to 10 DPI (Supplementary Table S1) (Chen et al., 2008). Groups receiving prophylactic supplementation of baicalin (CD+BC1 and CD+BC2) had a significantly reduced average clinical score compared with the challenge control (CD) (*p* < 0.05) (Figure 2a). The recovery of surviving morbid animals in the *C. difficile* control group was much slower compared with baicalin-treated groups (*p* < 0.05), with apparent clinical resolution observed by 9 DPI. However, the baicalin-supplemented groups showed a dose-dependent reduction in disease severity, with complete recovery observed by 6 DPI (*p* < 0.05). Although not statistically significant, the clinical score of the CD+BC2 group was lower compared with that of CD+BC1 group. Interestingly, a similar trend in the average clinical scores was also observed in the baicalin therapeutic trial. The clinical scores in CD+BC1 and CD+BC2 groups also followed a dose-dependent reduction in disease severity (Figure 2b). However, the recovery rate was much slower compared with the prophylactic study, where no complete resolution of clinical disease was observed until the end of the experiment (day 10 post-*C. difficile* challenge).

Body weights were recorded on a daily basis post infection, and the relative percentage weight with respect to the initial weight prior to the *C. difficile* challenge was calculated. In the prophylactic study, the baicalin control group (BC2) and Ant+BC2 group showed no significant weight loss compared with negative control. However, mice in the *C. difficile*-positive control (CD) showed a significant and progressive weight loss from 1 DPI to 5 DPI compared with unchallenged control (*p* < 0.05), with animals regaining their pre-challenge body weights by 9 DPI. Although there was no significant difference in the average body weights of mice from the BC-treated challenge groups (CD+BC1 and CD+BC2) compared with positive control, baicalin-treated animals were able to rapidly regain their pre-challenge body weights by 5 DPI compared with the *C. difficile*-positive control (9 DPI) (Figure 2c).

In the therapeutic trial, *C. difficile*-challenge control (CD) showed significant weight loss compared with negative controls (*p* < 0.05). Mice in the *C. difficile*-challenge control group showed progressive weight reduction 3 DPI through 6 DPI, returning to their initial body weights by 8 DPI. In addition, there was no significant difference in average percentage body weights between the CD group and CD+BC1 group. However, a significant difference was observed in the average percentage body weights of the CD+BC2 groups compared with the CD group on days 3 and 4 post challenge (*p* < 0.05) (Figure 2d). Moreover, the CD+BC2 group attained their pre-challenge body weight by 4 DPI; however, a slight delay was observed in the CD+BC1 group, with animals attaining their initial body weight by 6 DPI (Figure 2d).

### 2.3. Effect of Baicalin Supplementation on the Gut Microbiome of C. difficile-Challenged and Non-Challenged Mice

**Figure 3:**
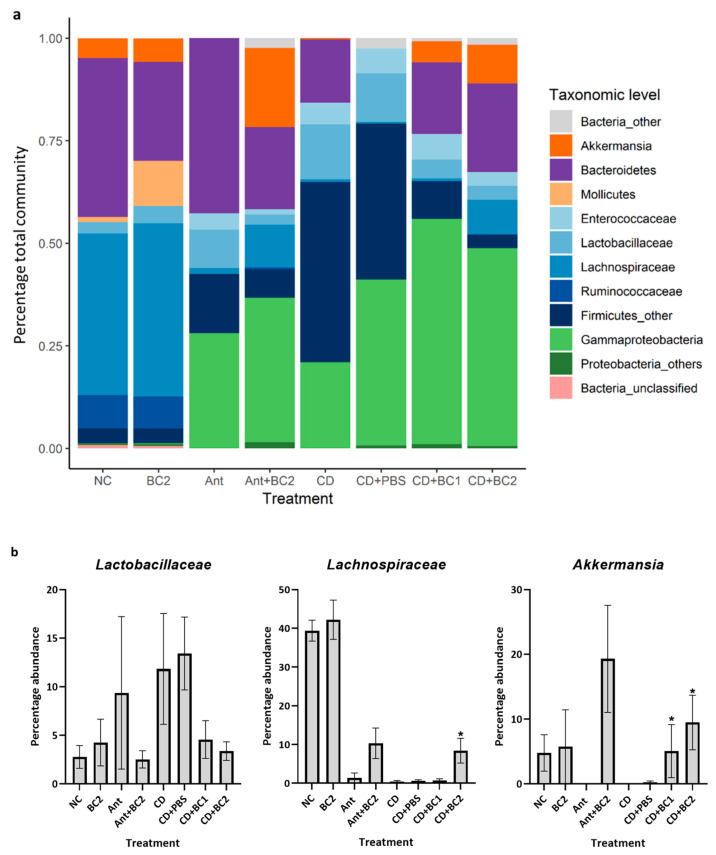

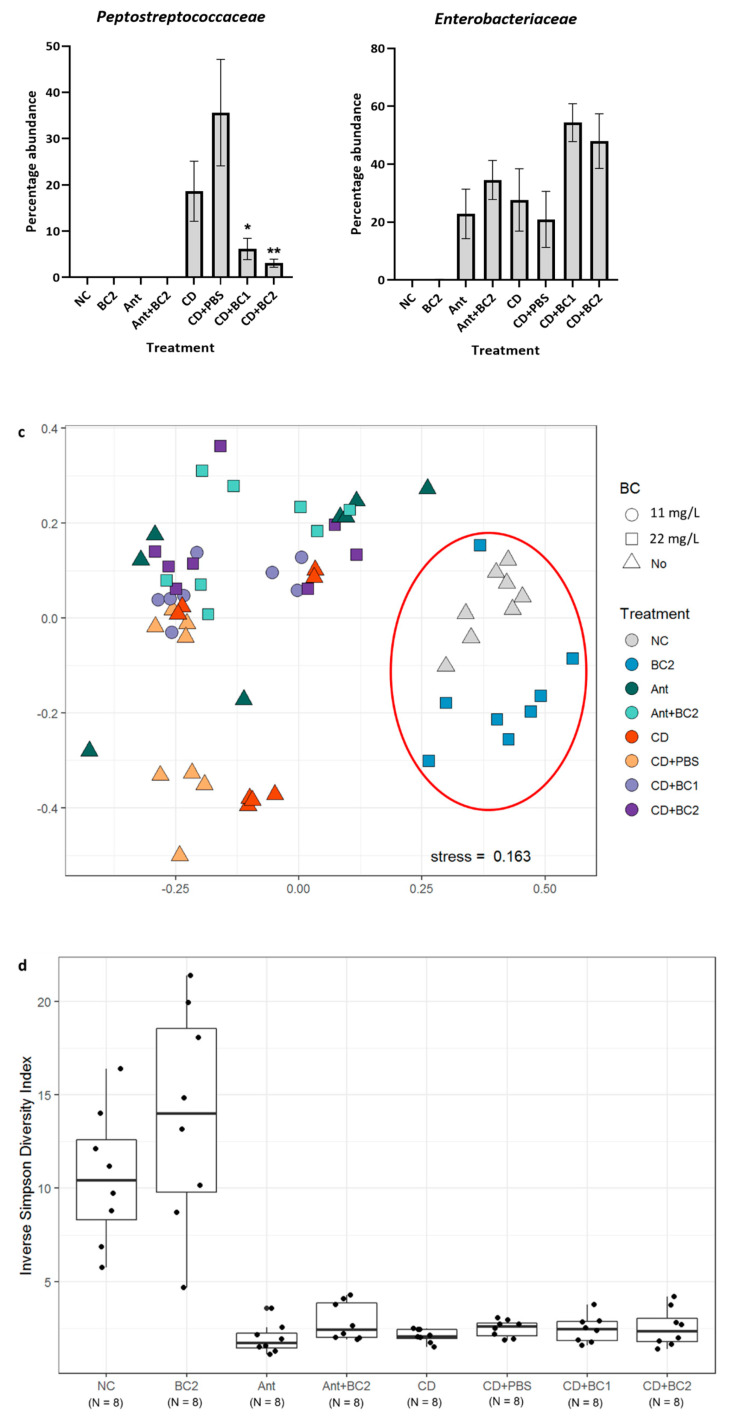
Effect of baicalin supplementation on the abundance of major gut microbiome taxa and microbiome diversity in the antibiotic-treated and *C. difficile*-challenged mice. (**a**) Relative taxa abundance of OTUs: Fecal samples were collected 2 DPI from the prophylactic BC study. DNA was extracted for microbiome analysis using Illumina MiSeq platform, and the relative abundance of OTUs of major phyla, order, family, and genera was determined. (**b**) Abundance of major bacterial family taxa. Percentage abundances of major families—*Lactobacillaceae*, *Lachnospriaceae*, *Akkermansia*, *Peptostreptococcaceae,* and *Enterobacteriaceae*—in the mice treatment groups of the prophylactic BC study. **, * indicates a statistically significant difference (*p* < 0.001, *p* < 0.05, respectively) relative to the untreated challenge group (CD). Error bars represent SEM. (**c**) Bray–Curtis plot: Relationships between treatment groups based on the abundance of species present in each sample were plotted. NMS ordinations were run in R (v 3.3.0) using metaMDS in the vegan (v 2.3-5) package after calculating the stress scree plots to determine the number of axes required to achieve stress below 0.2, plotted using ggplot2 (v 2.1.0). (**d**) Inverse Simpson Plot: Fecal samples were collected 2 DPI of the prophylactic BC study. DNA was extracted for microbiome analysis using Illumina MiSeq platform, and Alpha diversity was calculated using inverse Simpson to measure the richness and evenness of the OTUs. Treatment groups: NC (unchallenged negative control), Ant (unchallenged antibiotic control), Ant+BC (unchallenged antibiotic + 22 mg/L BC control), BC2 (unchallenged 22 mg/L BC control), CD (Ant+*C. difficile*-challenged control), CD+PBS (Ant+*C. difficile*-challenged control, PBS solvent control), CD+BC1 (Ant+CD + 11 mg/L BC), CD+BC2 (Ant+CD + 22 mg/L BC).

The results from the prophylactic trial revealed distinctive patterns in the composition of bacterial communities in the different treatment groups. In the unchallenged control group (NC), the predominant phyla groups consisted of Firmicutes and Bacteroidetes in a ratio of 1.05:1, with a minimal proportion of other taxa related to opportunistic pathogens such as Gammaproteobacteria and *Enterococcaceae* (Figure 3a). In the baicalin control group (BC2), a higher proportion of Firmicutes was observed, compared with Bacteroidetes having a ratio of 1.79:1. Although, the phyla comparisons seemingly had a greater degree of difference in their proportion across groups, it was statistically insignificant. The antibiotic control group (Ant) had a higher proportion of Gammaproteobacteria and *Enterococcaceae* compared with the negative control and baicalin control group. The supplementation of baicalin along with the antibiotic (Ant+BC2) seemed to reduce the proportion of *Enterococcaceae* but was not able to reverse the increase in Gammaproteobacteria. However, there was an increase in the proportion of the phylum Verrucomicrobia (represented as genus *Akkermansia*) compared with the antibiotic control group (Figure 3a). The baicalin untreated challenge groups (CD and CD+PBS) had a predominantly higher proportion of Firmicutes and Gammaproteobacteria compared with uninfected controls. However, baicalin administration to *C. difficile*-challenged groups (CD+BC1 and CD+BC2) reduced the abundance of Firmicutes and increased the proportion of Proteobacteria compared with the antibiotic control and positive control groups (CD and CD+PBS). A notably distinct phylum that prevailed among baicalin-treated, spore-challenged (CD+BC1 and CD+BC2), and unchallenged (BC2 and Ant+BC2) groups was Verrucomicrobia, specifically the genus *Akkermansia* (Figure 3a).

At the family/genus level, the relative abundance of *Lactobacillaceae* did not show any significant difference amongst the negative control (NC), baicalin control (BC2), and baicalin-treated antibiotic control groups (Ant+BC2) (*p* > 0.05). In contrast, the antibiotic control and untreated spore challenge groups (CD and CD+PBS) had a higher abundance of *Lactobacillaceae* compared with the aforementioned controls; however, it was not statistically significant. However, although not significant, baicalin-treated spore challenge groups had a much lower abundance of *Lactobacillaceae* compared with positive controls (Figure 3b). With regards to *Lachnospiraceae* and *Akkermansia*, although not significant, baicalin-treated control (BC2) marginally increased their relative abundance compared with the negative control (*p* > 0.05) (Figure 3b). In untreated spore challenge groups (CD and CD+PBS), the abundance of both *Lachnospiraceae* and *Akkermansia* was significantly reduced compared with the negative control (NC), baicalin control (BC2), and the baicalin-treated antibiotic control (Ant+BC2) (*p* < 0.05). However, with the exception of the CD+BC1 group, there was a significant increase in the relative abundance of *Lachnospiraceae* in the CD+BC2 group compared with untreated spore challenge groups (CD and CD+PBS) (*p* < 0.05). In terms of the relative abundance of *Akkermansia*, there was a significant increase in both baicalin-treated challenge groups compared with untreated spore challenge groups (*p* < 0.05) (Figure 3b). The relative abundance of *Peptostreptococcaceae* was negligible and showed no significant difference in the negative control (NC), baicalin control (BC2), and antibiotic controls (Ant and Ant+BC2 groups) (*p* > 0.05) (Figure 3b). However, in baicalin-treated spore-challenged groups (CD+BC1 and CD+BC2), *Peptostreptococcaceae* was significantly reduced compared with the CD+PBS group (not CD group), which had a higher abundance (*p* < 0.05). The abundance of *Enterobacteriaceae* was higher in antibiotic control (Ant), baicalin-treated antibiotic control group (Ant+BC2), *C. difficile-*positive control and PBS control (CD and CD+PBS) groups compared with negative control and baicalin control (BC2) groups (Figure 3b).

The non-metric multi-dimensional scaling (NMDS) plot indicating the differential pattern of bacterial diversity revealed a close clustering of baicalin control (BC2) and negative control, suggesting that the species abundance in the BC2 group is comparable with the untreated negative control. However, the other treatment groups (antibiotic-treated groups, challenged or unchallenged with *C. difficile*, and with or without BC treatment) did not indicate a typical relationship pattern for the abundance of species present in each sample (Figure 3c). The inverse Simpson plot representing the differential pattern of bacterial diversity revealed that the BC2 group did not alter the diversity of the gut bacterial community compared with the negative control (NC) (*p* > 0.05). However, irrespective of the baicalin treatment, there was a marked reduction in the diversity of bacterial communities in *C. difficile*-infected groups and antibiotic controls (Figure 3d).

### 2.4. Effect of Baicalin Supplementation on Histopathologic Lesion Score of C. difficile-Infected and Non-Infected Mice

**Figure 4:**
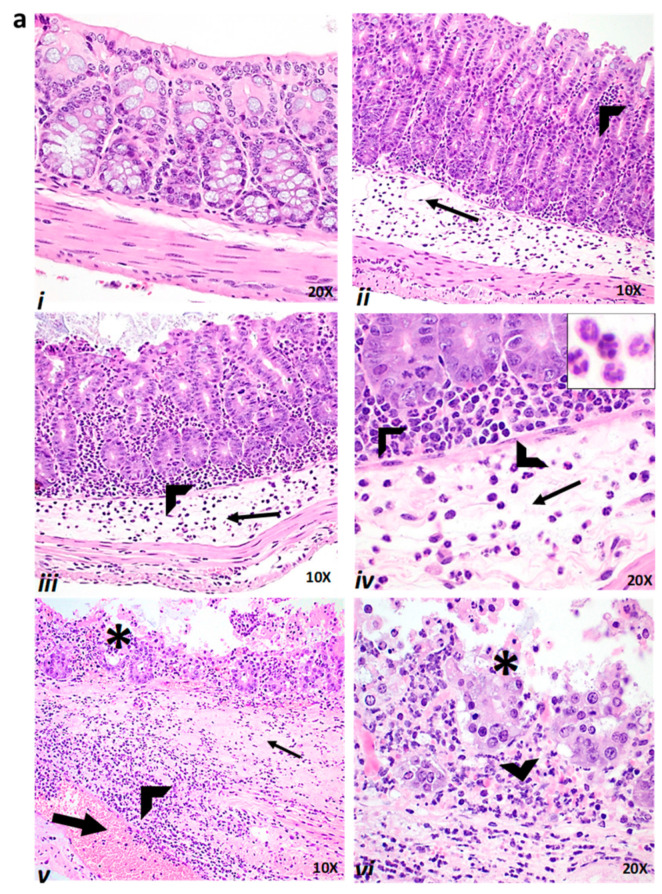

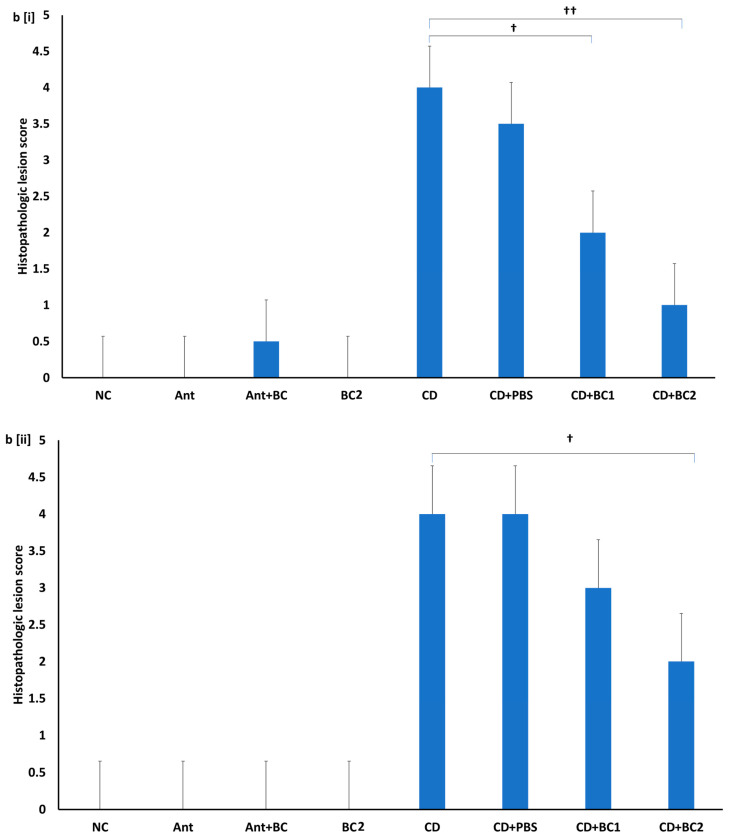
Colon histology and lesion scores. (**a**) Histologic examination of representative colonic tissues; (i) NC group—normal colon appearance; (ii) CD+BC1 group—colitis with submucosal and lamina propria inflammation (arrowhead) and moderate submucosal edema with dilated lymphatics (arrow); (iii,iv) CD+BC2 group—moderate colitis with submucosal edema (arrow), inflammation composed predominantly of neutrophils (arrowhead), and inset in (iv) shows neutrophils in higher magnifications (60×), which are predominantly inflammatory cells observed in the lamina propria and submucosa; (v,vi) CD group—severe colitis with marked submucosal protein-rich edema (arrow) congestion (thick arrow) and hemorrhage, enterocyte necrosis (asterisk) and erosion, and marked neutrophil infiltration (arrowhead). (**b**) Histopathologic scoring was based on (1) epithelial tissue damage; (2) congestion, edema, and hemorrhage; and (3) neutrophil infiltration. A score of 0–4 was assigned to each animal, denoting 0 for the absence of lesion, 1 for minimal, 2 for mild, 3 for moderate, and 4 for the severe histopathologic lesion. Mean of individual category scores were calculated to provide an overall histopathologic lesion score for each mouse and then for each group. Error bars represent SEM. (i) Prophylactic study; (ii) therapeutic study. ††, † symbol indicates a statistically significant difference (*p* < 0.001, *p* < 0.05, respectively) relative to challenged, positive control (CD) vs. the baicalin-treated challenged mice (CD+BC1 or CD+BC2).

In both the prophylactic and therapeutic studies, the *C. difficile*-positive control (CD) and CD+PBS group showed significantly severe colitis compared with the unchallenged negative controls (*p* < 0.0001). The representative histopathological slides shown in the figure are from the treatment groups NC (Figure 4a(i)), CD+BC1 (Figure 4a(ii)), CD+BC2 (Figure 4a(iii,iv)), and CD (Figure 4a(v,vi)) and from the histopathological scores for all the treatment groups provided in Figure 4b(i,ii)). The microscopic observations and histopathological scores indicate that the model was a severe disease challenge model, and the positive and negative control treatment groups worked accordingly.

Interestingly, mice groups receiving the prophylactic supplementation of baicalin (CD+BC1 and CD+BC2) had a significantly reduced histopathologic lesion score compared with *C. difficile*-positive control (CD) (*p* < 0.05 and *p* < 0.001) (Figure 4a(ii–iv),b(i)). However, in the therapeutic study, only high baicalin dose treatment (CD+BC2) had significantly reduced histopathologic lesion score (*p* < 0.05) compared with *C. difficile*-positive control (CD) (Figure 4b(ii)). However, in both study designs, i.e., the prophylactic and therapeutic studies, the higher doses in the CD+BC2 group had decreased histopathologic lesions as compared with the low dose CD+ BC1, although this reduction was not statistically significant.

### 2.5. Effect of Baicalin Supplementation on Fecal C. difficile Counts and Fecal Toxin-Mediated Cytotoxicity on Vero Cells

**Figure 5:**
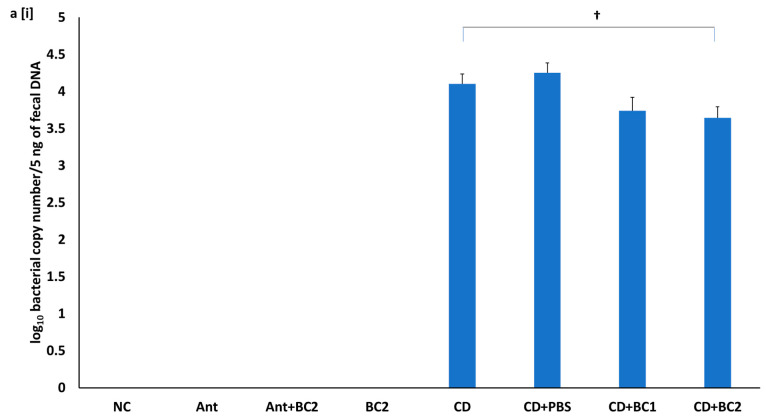

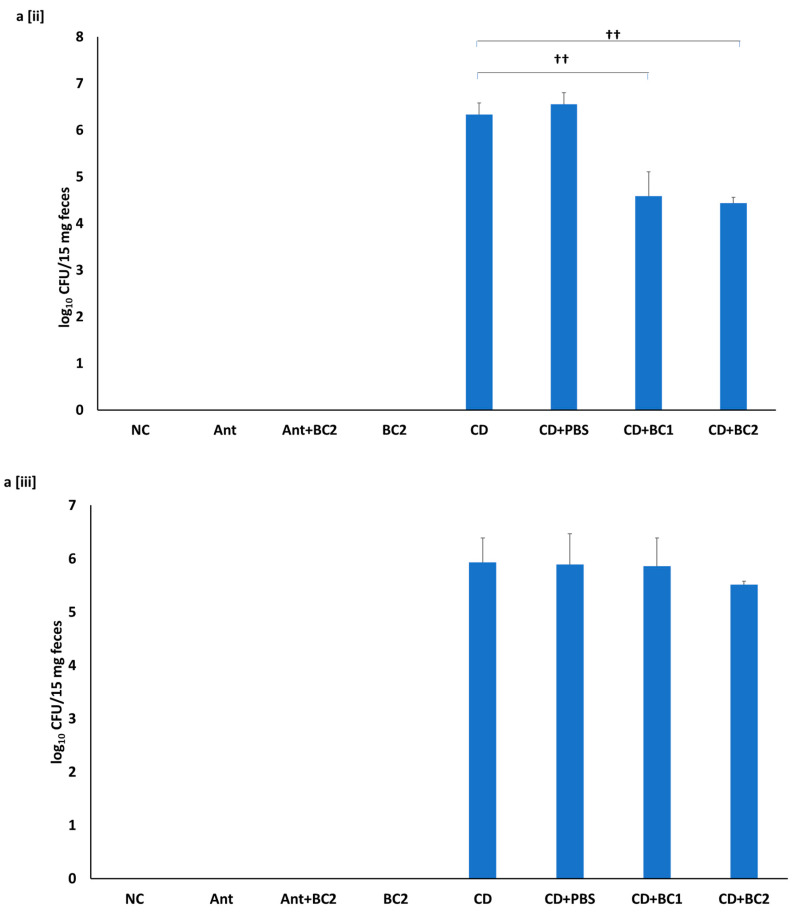

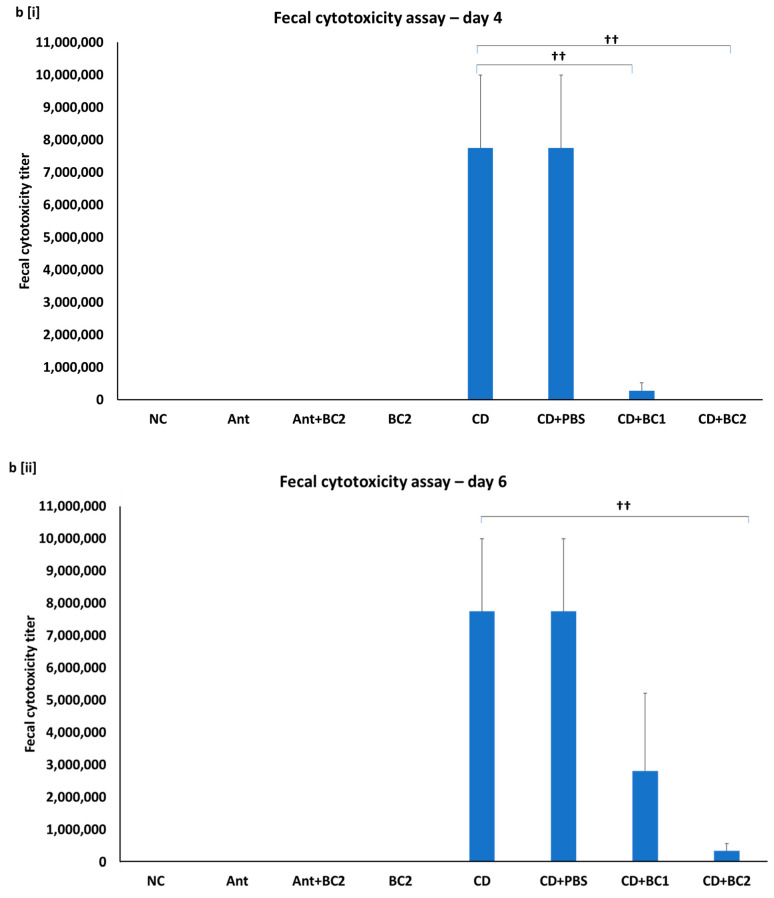
Effect of prophylactic baicalin supplementation on *C. difficile* counts and Vero cell cytotoxicity using fecal slurry supernatants. (**a**) Enumeration of *C. difficile* from fecal samples using (i) qPCR-based quantification using fecal DNA (5 ng content) from day 2 post infection, and (**a** (ii,iii)) serial dilution and plating of fecal samples on cycloserine-cefoxitin fructose containing 0.1% sodium taurocholate (CCFA-T) agar plates from day 4 and 6 post infection. (**b**(i,ii)) Fecal cytotoxicity assay using fecal samples collected during day 4 and 6 of the prophylactic study. Error bars represent SEM. ††, † symbol indicates a statistically significant difference (*p* < 0.001, *p* < 0.05, respectively) relative to challenged positive control (CD) vs. the baicalin-treated challenged mice (CD+BC1 or CD+BC2).

qPCR-based fecal *C. difficile* counts in the untreated, challenged mice groups, CD and CD+PBS, were 4.25 and 4.10 log CFU/mL, respectively. However, there was a mild reduction in counts by ~0.5 log CFU/mL in both the CD+BC1 and CD+BC2 treatment groups when compared with the untreated challenged mice groups (Figure 5a(i)). Fecal *C. difficile* spore enumeration was performed by serial dilution and plating on samples collected from day 4 and 6 post infection. Although there was approximately a 2-log reduction in *C. difficile* spore counts in the day 4 fecal samples of challenged, BC-treated mice compared with the positive control (CD) (*p* < 0.001), the *C. difficile* counts were almost the same level by day 6 post infection (*p* > 0.05) (Figure 5b(ii,iii)). In addition, the fecal slurry supernatants from the BC-treated, challenged mice showed a reduction in Vero cell cytotoxicity compared with the untreated, challenged mice. Vero cell cytotoxicity with day 4 and day 6 fecal samples from CD+BC1 and CD+BC2 mice groups showed 96% (*p* < 0.05) and 99% (*p* < 0.05), and 64% (*p* = 0.18) and 96% (*p* < 0.05) reduction, respectively when compared with the untreated, *C. difficile*-challenged mice (Figure 5b(i,ii)).

## 3. Discussion

In the current study, we investigated the prophylactic and therapeutic efficacies of baicalin as an alternative agent to ameliorate CDI without compromising the normal gut microbial population. Previous research conducted in our laboratory revealed that sub-inhibitory concentration of baicalin reduced *C. difficile* toxin production and cytotoxicity in vitro. Additionally, baicalin inhibited *C. difficile* spore germination and outgrowth [31]. The results from the current study translate our previous findings in vivo by demonstrating a dose-dependent reduction of CDI severity in BC-supplemented mice. Concurring with the reduced incidence of diarrhea in baicalin-treated *C. difficile*-infected mice (*p* < 0.05) (Figure 2a,b), a significant reduction in average clinical scores, fecal toxin-mediated Vero cell cytotoxicity, and histopathologic lesion scores were also observed compared with the challenge control group (CD) (*p* < 0.05) (Figure 2c,d, Figure 4b and Figure 5b). Although the fecal *C. difficile* counts on day 6 were comparable across the challenged mice groups treated with or without BC (5a (iii)), there was a significant reduction in the fecal toxin-mediated Vero cell cytotoxicity in the CD+BC2 mouse group (Figure 5b(ii)). The reduced CDI severity in baicalin-treated mice could be attributed to the inhibitory effect of baicalin on *C. difficile* toxin production, as observed in our in vitro studies [31]. In addition, baicalin is known to possess anti-inflammatory and anti-diarrheal properties [34,35,36], which could also have contributed to the improved clinical outcome in BC-administered mice.

A normal and healthy gastrointestinal microbiota is key for preventing pathogen colonization, including *C. difficile* [37]. Disruption of host gut microbiota as a result of antibiotic therapy is the most important predisposing factor for CDI [1]. Antibiotic administration significantly alters microbiome diversity and composition, the effects of which can persist even after the withdrawal of antibiotics [38,39]. The increased risk for CDI susceptibility in the elderly is attributed to the reduction of the protective bacterial population such as Firmicutes and undesirable Proteobacteria groups in the gut [40,41,42].

In this study, baicalin did not reduce the bacterial diversity of the mouse gut microbiome compared with the untreated negative control (Figure 3c,d). Baicalin treatment alone significantly increased the abundance of Firmicutes, especially the members of *Lachnospiraceae* and, to a modest extent, the *Lactobacillaceae* group, compared with the negative control (Figure 3b). Microbiome analyses of human CDI patients by previous researchers have identified that *Ruminococcaceae* and *Lachnospiraceae*, as well as butyrate-producing bacteria were significantly depleted in patients with CDI compared with healthy subjects, whereas *Enterococcus* and *Lactobacillus* were more abundant in CDI patients [43]. In addition, a decrease in *Enterococcaceae* along with an increase in *Peptostreptococcaceae, Lactobacillaceae,* and *Enterobacteriaceae* have also been reported in *C. difficile*-positive patients [44,45,46,47,48,49]. An abundance of *Lachnospiraceae*, *Ruminococcaceae*, and *Bacteroidaceae* families mainly contribute to *C. difficile* colonization resistance in humans [43,47,50]. Similar observations in the gut microbiome of mice were also observed, wherein an increase in *Lactobacillaceae* and *Enterobacteriaceae* families was noted in susceptible mice that were treated with antibiotics, whereas *Lachnospiraceae* dominated in animals that remained resistant to CDI [51]. In addition, it has been collectively implicated from several research findings that that a decrease in *Lachnospiraceae* and Barnesiella with an increase in *Lactobacillaceae* and *Enterobacteriaceae* is responsible for the loss of colonization resistance against *C. difficile* [50]. Antibiotic-induced microbiome dynamics observed in the current study are in agreement with the findings reported by previous researchers. Antibiotic pre-treatment significantly increased the abundance of the *Lactobacillaceae* and Proteobacteria, with a drastic reduction in the *Lachnospiraceae* (Figure 3b). This change in the microbial composition could be correlated with an increased susceptibility of mice to *C. difficile* challenge. *Akkermansia* genus (phylum Verrucomicrobia) is a strictly anaerobic, Gram-negative bacterium that has been detected in the intestine of most healthy individuals, representing 1–4% of the total microbiota, and is capable of utilizing gut-secreted mucin as a sole source of carbon and nitrogen [52,53]. The only species in this genus, *Akkermansia muciniphila*, has beneficial effects on metabolism and gut health by exhibiting anti-inflammatory and immunostimulant properties [53,54]. Recent studies have revealed that co-administration of *A. muciniphila* with polyphenols or prebiotics resulted in improvement of gut barrier function and reduced endotoxemia [55]. Although co-administration of baicalin with antibiotics (Ant+BC2) was not able to reverse the abundance of *Enterobacteriaceae*, a significant increase in *Lachnospiraceae* and *Akkermansia* was observed (*p* < 0.05) (Figure 3b). Therefore, in the CD+BC1 and CD+BC2 groups, the microbiome shift observed during co-administration of baicalin and antibiotics may have contributed to the colonization resistance against *C. difficile* on days 2 and 4 post infection. The untreated spore-challenged mice groups (CD and CD+PBS) had invariably shown an increased abundance of *Lactobacillus* and Proteobacteria due to antibiotic administration, along with an increase in the abundance of *Peptostreptococcaceae*, the family under which the pathogenic *C. difficile* are classified [56]. However, in baicalin-treated spore challenge groups, we observed a dose-dependent increase in the abundance of *Lachnospiraceae* and *Akkermansia,* along with a significant reduction in *Peptostreptococcaceae* (*p* < 0.05) (Figure 3b). These results suggest that the reduced clinical symptoms and infection in baicalin-treated animals could be attributed in part to the beneficial shift in the gut microbiome, especially with the improved abundance of *Lachnospiraceae* and *Akkermansia*.

## 4. Materials and Methods

### 4.1. Ethics Statement, Animals, and Housing

The study was performed with the approval of the Institutional Animal Care and Use Committee (IACUC) at the University of Connecticut, following the endorsed guidelines for animal care and use. Five- to six-week-old C57BL/6 mice were obtained from Charles River (Boston, MA), housed in a biohazard level II AALAC-accredited facility, and monitored for health status twice daily. Mice were provided with irradiated feed, autoclaved water, and bedding, along with 12 h light/dark cycles. The procedures that required animal handling (spore administration, cage changes, and sample collection) were done under a biosafety cabinet (class II) using proper personal protective equipment. Decontamination and sterilization of the biosafety cabinet was done using 10% bleach to prevent cross-contamination between experimental treatment groups. The mice were singly housed in a cage, and twelve cages were included for each treatment in each of the experiments.

### 4.2. Prophylactic and Therapeutic Administration of Baicalin in a Mouse Model of C. difficile Infection

The in vivo infection model was based on a previously established protocol with minor modifications [32]. Five- to six-week-old female animals were randomly assigned to one of the following eight treatment groups of thirteen animals each (Table 1). In the prophylactic model, animals were provided irradiated pellet feed and incorporated baicalin in drinking water containing 0, 11, and 22 mg/L of the compound for a period of twenty-two days (Supplementary Figure S1). As equated from the average daily water consumed by each mouse (~5–7 mL per day), baicalin-treated water was expected to deliver approximately 250 mg/kg and 500 mg/kg of the compound per day in the 11 and 22 mg/L treatments, respectively. Previous researchers have indicated that baicalin dosage of 400 mg/kg is well tolerated by mice [57]. Subsequently, an antibiotic cocktail comprising kanamycin (0.4 mg/mL), gentamicin (0.03 mg/mL), colistin (850 U/mL), metronidazole (0.215 mg/mL), and vancomycin (0.045 mg/mL) was added in drinking water for 3 days. After antibiotic supplementation, the mice were switched back to their prior treatment regimens, and all animals in the challenge groups (CD, CD+PBS, CD+BC1, and CD+BC2) and the antibiotic control group (Ant) received a single intraperitoneal injection of clindamycin (10 mg/kg, with a maximum of 0.5 mL/mouse using a 27G needle and syringe) a day prior to *C. difficile* challenge. Pre-treatment of mice with antibiotics was intended to induce gastrointestinal dysbiosis and enable *C. difficile* colonization following the spore challenge. Mice proposed for *C. difficile* infection were orally administered 10^6^ spores (CFU) per 0.1 mL total volume of hypervirulent *C. difficile* ATCC BAA 1803 using a straight 18G gavage needle (1” shaft length) and were observed for signs of CDI, including diarrhea, wet tail, and hunched posture using a mouse clinical score sheet (Supplementary Figure S2 and Supplementary Table S1).

The individual weight of each mouse was measured every day, fecal samples were collected on alternate days post infection (DPI; days 4 and 6 for the prophylactic study only), and all animals were observed twice daily for ten days for morbidity and mortality. At the end of the experiment (10th day after challenge), all animals were euthanized. In the therapeutic model, the only difference from the aforementioned procedure is that baicalin was administered from day 1 post-*C. difficile* spore challenge (1 DPI). In addition, microbiome analysis was not performed in the therapeutic study (Supplementary Figure S1).

### 4.3. Histopathologic Analysis

Colon and cecum were collected from each mouse from the prophylactic study (*n* = 8), and the tissues were fixed in 10% formalin. Formalin-fixed tissues were then embedded in paraffin, and slides were made and stained with hematoxylin and eosin. A board-certified veterinary pathologist performed blinded histopathological analysis on all sections. Histopathologic grading was based on a scoring system reported previously [32,58]. Briefly, scoring was based on (1) epithelial tissue damage; (2) edema, congestion, and hemorrhage; and (3) neutrophil infiltration. A score of 0–4 was assigned to each animal, with 0 denoting the absence of lesion, 1 for minimal, 2 for mild, 3 for moderate, and 4 for severe. Mean of individual category scores were calculated to provide an overall histopathologic lesion score for each mouse and then for each group.

### 4.4. DNA Extraction, PCR Amplification, and Sequencing of Taxonomic Markers

Fecal samples from day 2 post infection from all treatment groups (from eight animals per treatment group) of the prophylactic baicalin study were subjected to DNA extraction using the MoBio PowerMag Soil 96 well kit (MoBio Laboratories, Inc., Carlsbad, CA, USA), according to the manufacturer’s protocol for the Eppendorf ep Motion liquid-handling robot. Quantification of DNA was performed using the Quant-iT PicoGreen kit (Invitrogen, ThermoFisher Scientific, Waltham, MA, USA), and DNA was subjected to amplification of partial bacterial 16S rRNA genes (V4 region) from 30 ng of extracted DNA as template, using 515F and 806R primers bound with Illumina adapters and dual indices (8 basepair golay in 3′ and 5′) [59,60]. Amplification was performed in triplicates with the addition of 10 µg BSA (New England BioLabs, Ipswich, MA, USA) using Phusion High-Fidelity PCR master mix (New England BioLabs, Ipswich, MA, USA). The reaction mixes were incubated at 95 °C for 3.5 min and then subjected to PCR reaction for 30 cycles of 30 s at 95.0 °C, 30 s at 50.0 °C, and 90 s at 72.0 °C, followed by a final extension at 72.0 °C for 1 min. Quantification and visualization of pooled PCR products were performed using the QIAxcel DNA Fast Analysis (Qiagen, Germantown, MD, USA). DNA concentrations of the PCR products were normalized to 250–400 bp and pooled using the QIAgility liquid handling robot. Pooled PCR products were cleaned up using the Gene Read Size Selection kit (Qiagen, Germantown, MD, USA) according to the manufacturer’s protocol, and the cleaned pool was subjected to sequencing on MiSeq using a v2 2 × 250 base pair kit (Illumina, Inc., San Diego, CA, USA).

### 4.5. Sequence Analysis

Microbiome analysis was set up as a completely randomized design with treatments done in replicates of eight. Filtering and clustering of sequences were performed using Mothur 1.36.1 based on a published protocol [60]. The operational taxonomic units (OTUs) of samples were clustered at 97% sequence similarity, and downstream analysis was done using R version 3.2. The richness and evenness of sample OTUs were calculated by estimating alpha diversity using inverse Simpson diversity index, which were then analyzed using Tukey’s test. Permutational multivariate analysis (PERMANOVA, adonis function, 75 permutations) was performed to analyze differences in bacterial community composition in the various treatment groups. Test for significance in alpha diversity was determined by ANOVA followed by Tukey’s honest significant differences, adjusting for multiple comparisons (*p* = 0.05). NMS ordinations were run in R (v 3.3.0) using metaMDS in the vegan (v 2.3-5) package after calculating the stress scree plots to determine the number of axes required to achieve stress below 0.2, plotted using ggplot2 (v 2.1.0). In addition, the relative abundance of OTUs of major phyla, order, and genera was determined to assess the effect of treatment. Tukey’s test was used to identify changes in groups of bacteria based on treatment, and the significance was detected at *p* < 0.05.

### 4.6. Fecal C. difficile Enumeration and Cytotoxicity Assay

DNA extracted from day 2 post-infection fecal samples in the previous section (prophylactic study; *n* = 8) was subjected to qPCR-based enumeration of *C. difficile*. The Ct values obtained were compared against the standard curve for the *tcd*A gene of BAA 1803. The bacterial counts for the standard curve ranged from 0.5 log CFU/mL to 6 log CFU/mL. The trendline from the scatter plot of the standard curve generated the regression equation *y* = −3.8456*x* = 36.4 (*R*^2^ = 0.9899), wherein *y* denotes the Ct values of the respective samples and *x* would provide the counts in log_10_ bacterial copy number/qPCR. Fecal material obtained from days 4 and 6 of the prophylactic study (*n* = 4) was subjected to *C. difficile* enumeration using serial dilution and plating. An amount of 15 mg of fecal material was weighed and transferred into an Eppendorf tube containing 500 µL of PBS. The samples were mixed thoroughly by vortexing and subjected to heat shock at 60 °C for 20 min (in a water bath) to kill the vegetative bacteria and favor sporulation of *C. difficile* in the fecal slurry. To enumerate *C. difficile* spores, the samples were serially diluted and plated on to cycloserine-cefoxitin fructose agar containing 0.1% sodium taurocholate (CCFA-T)) and incubated in an anaerobic chamber (A35, Don Whitley Scientific Ltd., Bingley, UK) at 37 °C for 48 h. In addition, fecal samples collected from day 4 and 6 post infection were also subjected to Vero cell cytotoxicity. In this method, 15 mg of fecal material was weighed and mixed thoroughly by vortexing with 200 µL of sterile PBS and subsequently centrifuged at 14,000× *g* at 4 °C for 10 min (samples were stored at −80 °C if not used immediately). The supernatants of the fecal slurry were subjected to Vero cell cytotoxicity assay [31]. Fecal slurry supernatants were serially diluted by 1:10 up to a dilution of 1:100,000,000 onto confluent Vero cell monolayers in 96-well microtiter plates. The cell culture plates were incubated in a carbon dioxide incubator (5% CO_2_) at 37 °C for 24 h and observed for cytopathic changes under an inverted microscope. Cytopathic changes were observed as Vero cell rounding, and the cytotoxicity titer was considered as the highest microtiter well dilution showing 80% cell rounding. The identified titer values were expressed as the reciprocal of the identified dilution.

### 4.7. Statistical Analysis

Data were analyzed using R and GraphPad Prism 8.4.2. Chi-squared test was used to compare diarrhea incidence rate between to different treatments. For analyzing the percentage body weight and average clinical scores, the differences between means between experimental groups across the days were compared by two-way mixed ANOVA using Tukey’s test. For analyzing the qPCR based fecal *C. difficile* counts and fecal cytotoxicity assay, the differences between means were compared using one-way ANOVA. Percentage abundance of major family taxa in the microbiome was analyzed using one-way analysis using the Mann–Whitney test. A two-sided Cochran–Armitage test was used to compare the histopathologic lesion scores between the groups, with the Benjamini and Hochberg correction being applied to *p* values. The statistical significance level was set at *p* < 0.05. Survival curve comparisons were analyzed using the log-rank (Mantel–Cox) test.

## 5. Conclusions

The results from this study suggest that oral BC supplementation protects mice from antibiotic-induced gut dysbiosis and CDI. Baicalin supplementation significantly reduced the incidence of diarrhea as well as the severity of CDI clinical symptoms and enteric lesions in mice. In addition, BC favorably modulated the composition of gut microbiota without detrimentally affecting the gut microbiome diversity. However, further mechanistic and clinical investigations are warranted to validate and extrapolate these results for controlling CDI in human patients.

## Figures and Tables

**Table 1:** Different treatment groups used in the experiment. Abbreviations: Ant (antibiotic); CD (*C. difficile*); BC (baicalin); PBS (phosphate buffered saline).

Group	Antibiotic	BC	Spore Challenge
NC (Unchallenged negative control)	-	-	-
Ant (Unchallenged antibiotic control)	+	-	-
Ant+BC (Unchallenged antibiotic + 22 mg/L BC control)	+	+	-
BC2 (Unchallenged 22 mg/L BC control)	-	+	-
CD (Ant + C. difficile challenged control)	+	-	+
CD+PBS (Ant + CD challenged, PBS solvent control)	+	-	+
CD+BC1 (Ant + CD + 11 mg/L BC)	+	+	+
CD+BC2 (Ant + CD + 22 mg/L BC)	+	+	+

## Supplementary Materials

The following are available online at https://www.mdpi.com/article/10.3390/antibiotics10080926/s1, Figure S1: Antibiotic-induced murine CDI model, Figure S2: Mouse body condition chart, Table S1: Mouse clinical score sheet, Figure S3: Mouse survival curves.
