# Supplementary material for: Protective Effect of Baicalin against Clostridioides difficile Infection in Mice
Source: Antibiotics (Basel). 2021 Jul 30;10(8):926. doi: 10.3390/antibiotics10080926 (PMC8388895; doi:10.3390/antibiotics10080926)
Supplement: Supplementary file 1 [file antibiotics-10-00926-s001.zip › antibiotics-1242390-supplementary.pdf]

Figure S1: Antibiotic-induced murine CDI model

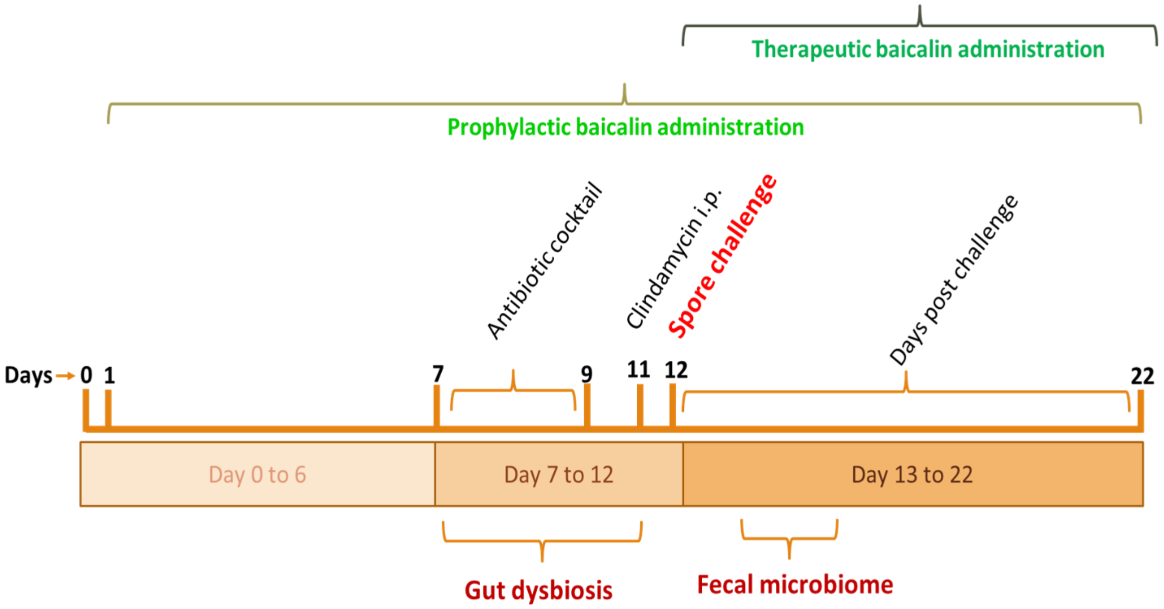

Figure S2: Mouse body condition chart

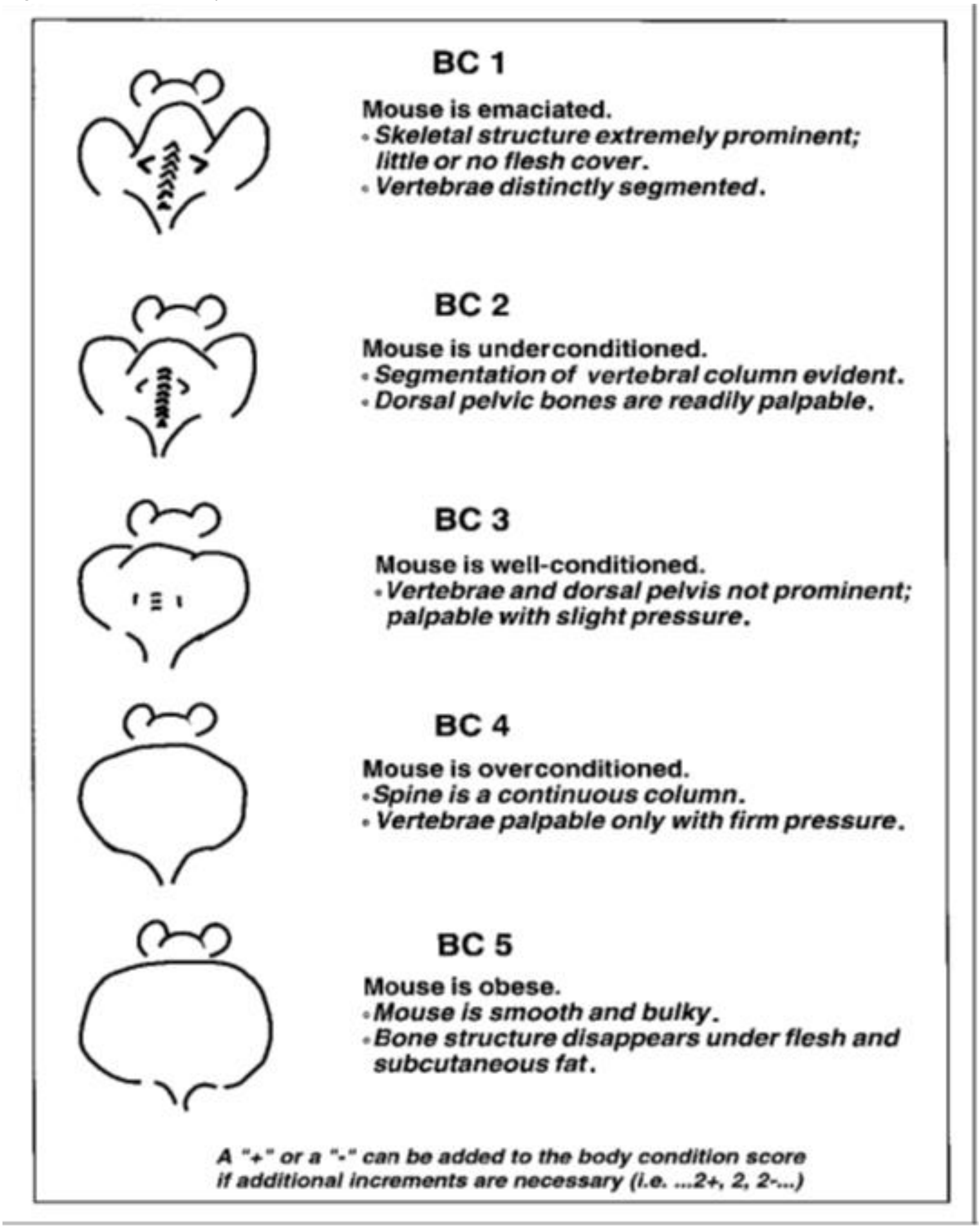

Figure S3: Mouse survival curves

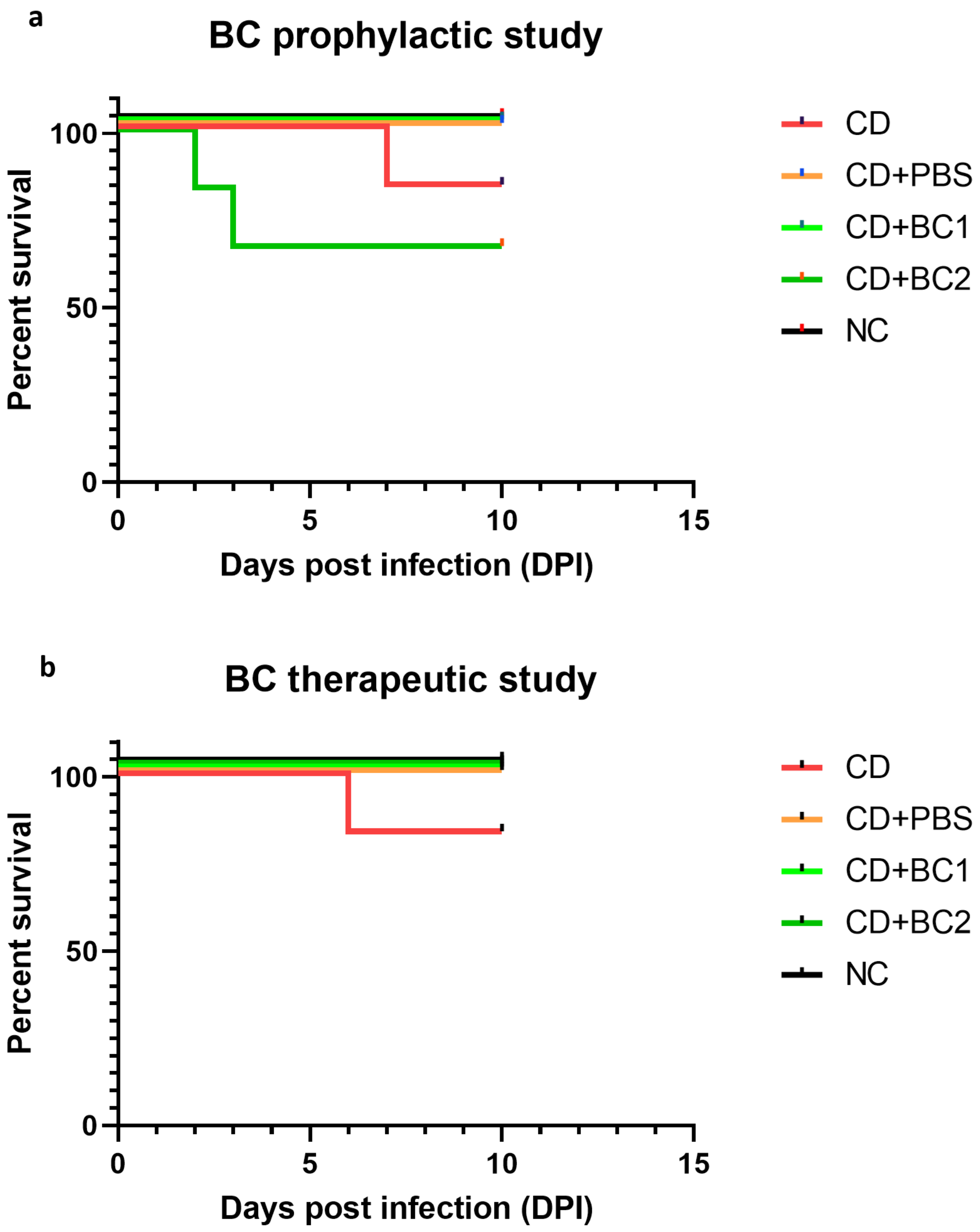

Table S1: Mouse clinical score sheet

MOUSE CLINICAL SCORE SHEET				
	0	1	3	5
Coat	Normal	Lack of grooming	Rough/Ruffled fur	
Activity	Normal	Isolated, abnormal posture	Huddled/inactive	Moribund, unresponsive or seizuring
Breathing	Normal	Rapid, shallow	Rapid, abdominal	Laboured, blue
Movement	Normal	Slight incoordination/ abnormality	Incoordinated, walking of toes, reluctant to move	Staggering, paralysis, lack of mobility
Condition	BC 3	BC 2+	BC 2	BC 1
Dehydration	Nil	Skin less elastic	Skin tents	Skin tents, eyes sunken
Feces	Normal	Moist faeces	Diarrhoea or dry faeces	Uncontrolled diarrhoea, Wet tail
Body Weight	Normal	Markedly reduced growth (e.g. severe runting) 5% over 24 hours	Weight loss up to 10% over 24 hours	Weight loss >10% over 24 hours or >20% over time

- Moribund animals (A lack of responsiveness to manual stimulation, lack of mobility or inability or failure to eat or drink or a clinical score > 25) will be euthanized.
